# Changes in hematoma volume following aneurysmal subarachnoid hemorrhage and its impact on patient prognosis

**DOI:** 10.3389/fneur.2024.1490957

**Published:** 2025-01-08

**Authors:** Zhenshan Huang, Feng Qian, Kui Ma, Guowei Jiang, Lianfu Zhang, Yongming Zhang

**Affiliations:** ^1^Department of Neurosurgery, Anhui No. 2 Provincial People’s Hospital, Hefei, Anhui, China; ^2^Anhui Medical University, Hefei, Anhui, China

**Keywords:** aneurysmal subarachnoid hemorrhage, 3D-Slicer, clinical prognosis, hematoma volume, neurosurgery

## Abstract

**Objective:**

This study aims to investigate the effects of preoperative intracerebral hematoma volume (HVpre), hematoma volume 6–8 days post-surgery (HVpost), and the rate of hematoma volume change (HVpre−HVpost)/HVpre on the prognosis of patients with aneurysmal subarachnoid hemorrhage (aSAH).

**Materials and methods:**

CT imaging data from 62 aSAH patients admitted to our hospital’s Neurosurgery Department between January 2022 and December 2023 were obtained, both preoperatively and 6–8 days postoperatively. The hematoma volumes were measured using 3D-Slicer. Patients’ recovery at 3 months post-discharge was assessed using the Modified Rankin Scale (mRS), categorizing the patients into a good prognosis group (mRS score 1–2) and a poor prognosis group (mRS score 3–5). Multivariate logistic regression analysis was conducted to identify independent risk factors for poor prognosis. Statistical methods were employed to compare preoperative and postoperative hematoma volumes with commonly used clinical scores. The predictive value of HVpre and HVpost for poor prognosis was evaluated using ROC curves. The rate of volume change was stratified by interquartile ranges, and the impact of different change rates on prognosis was compared.

**Results:**

Significant differences were found between good and poor prognosis groups in age, GCS score, Hunt-Hess grade, mFisher grade, BVpre, BVpost, and (HVpre−HVpost)/HVpre (*p* < 0.05). Logistic regression identified gender, age, BVpre, BVpost, and volume change rate as independent risk factors (*p* < 0.01). Increased GCS scores and higher Hunt-Hess and mFisher grades correlated with increased HVpre and HVpost. Higher hemorrhage reduction rates were linked to better outcomes. ROC curves showed HVpre and HVpost AUC values (0.831 and 0.857, respectively) were significantly higher than clinical scales. An HVpre volume over 22.25 mL and HVpost over 15.67 mL indicated a higher risk of poor prognosis, with sensitivities of 79.3 and 80.7%, and specificities of 67.1 and 69.3%.

**Conclusion:**

HVpre, HVpost, and (HVpre−HVpost)/HVpre can serve as neuroimaging biomarkers for assessing patients after aSAH and can effectively predict clinical prognosis.

## Introduction

Subarachnoid hemorrhage (SAH) is a severe neurological condition primarily caused by the rupture of intracranial aneurysms. Research indicates that 9 out of every 100,000 people in the United States, and nearly 600,000 people worldwide each year, experience this condition (1). Despite advances in diagnostic and therapeutic techniques that have reduced mortality rates, most survivors still suffer from mental health issues, cognitive impairments, and permanent disabilities, significantly diminishing their quality of life. Furthermore, about half of the aneurysm ruptures occur in individuals younger than 55, leading to productivity losses and substantial social costs in this age group (2).

Endovascular treatment is commonly used for aSAH, but it does not reduce the volume of hemorrhage that has already occurred. Blood leaking into the subarachnoid space, ventricles, or brain parenchyma can lead to a range of local and systemic symptoms, including hydrocephalus, delayed cerebral infarction, and cerebral vasospasm. These symptoms often result in secondary brain injuries, severely impacting patient prognosis (3–5). Currently, the Fisher scale or modified Fisher scale (mFS) is typically used to qualitatively assess the hematoma volume in aSAH. However, the mFS is susceptible to evaluator bias, and even with semi-quantitative methods, accurate quantification of hemorrhage is challenging (6). Therefore, an objective and precise measurement of hematoma volume in the subarachnoid space, brain parenchyma, and ventricles following intracranial aneurysm rupture, coupled with the establishment of a quantitative assessment system for aSAH hematoma volume, could potentially improve the accuracy of prognosis predictions for patients.

3D-Slicer is a free, open-source software designed for medical image processing, analysis, and visualization, and it can be used to accurately measure the volume of irregular intracranial hematomas (7). Utilizing 3D-Slicer to quantitatively measure hematoma volumes in the subarachnoid space and ventricles after aSAH holds significant potential for establishing a quantitative evaluation system to predict patient prognosis. Additionally, given that few studies have explored the impact of blood volume changes approximately 1 week after aSAH on prognosis, this study retrospectively collected CT imaging data from aSAH patients at admission and 6–8 days post-surgery. The hematoma volumes at both time points were measured using 3D-Slicer, and statistical methods were used to analyze their clinical value in predicting prognosis, with commonly used clinical scales serving as a comparison. The details of the study are reported as follows.

## Materials and methods

### Patients

**Figure 1:**
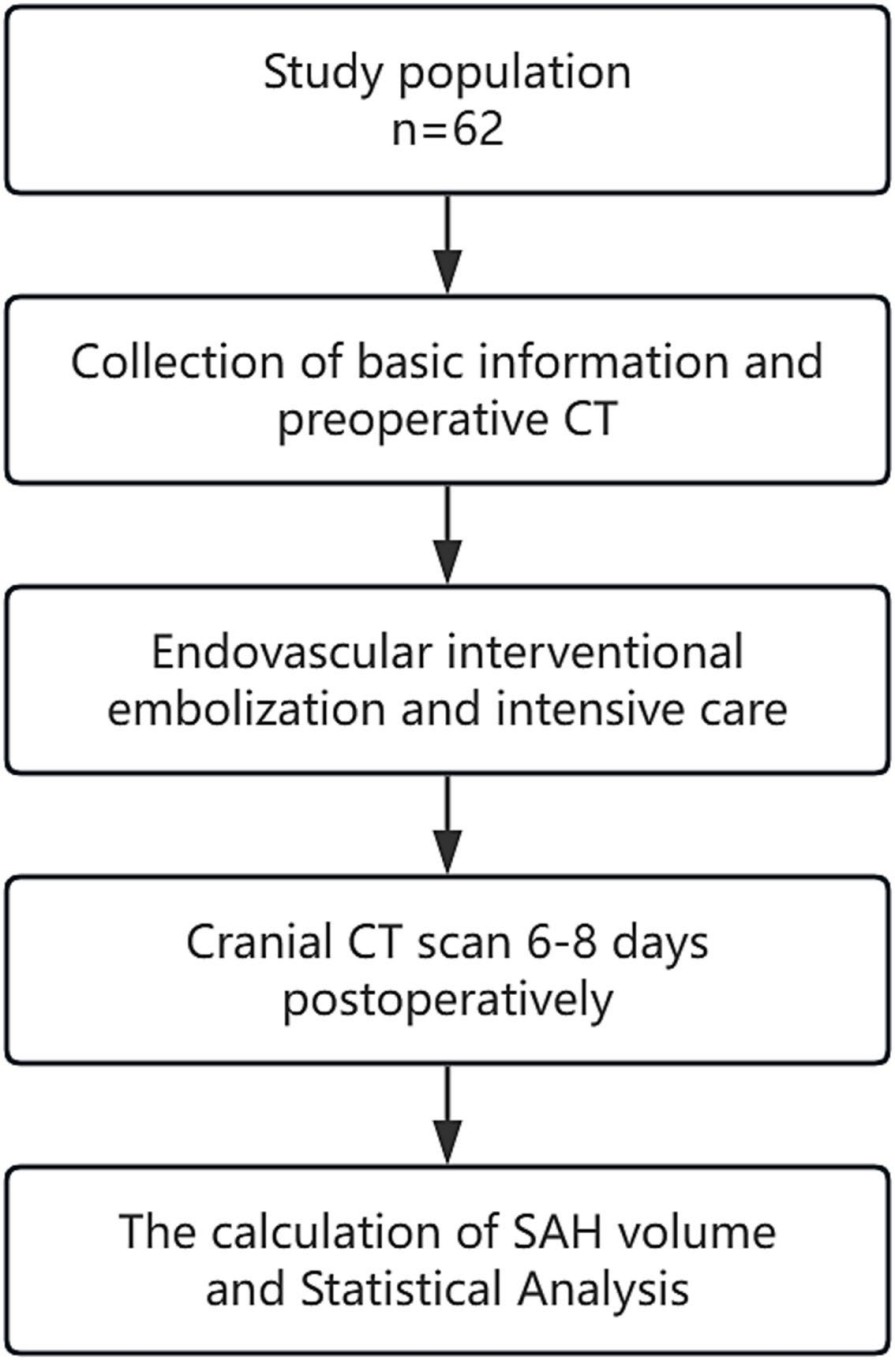
Flow chart.

A retrospective analysis was conducted on the clinical data of aSAH patients admitted to the Neurosurgical Intensive Care Unit of Anhui No. 2 Provincial People’s Hospital between January 2022 and December 2023. The flow chart is shown in Figure 1. All included patients were diagnosed with aSAH through cranial CT, CTA, and/or DSA and underwent early interventional surgery within 48 h of onset, followed by a cranial CT scan 6–8 days postoperatively. The exclusion criteria were: ① Patients with SAH caused by arteriovenous malformation rupture, trauma, or moyamoya disease; ② Patients with a history of stroke, intracranial tumors, or intracranial infections leading to neurological deficits; ③ Patients with coagulation disorders such as thrombocytopenia or hepatitis; ④ Patients with severe heart, liver, kidney, or lung diseases or organ failure; ⑤ Patients with Hunt-Hess grade V or postoperative rebleeding. This study was approved by the hospital’s Ethics Review Committee.

### Intravascular interventional embolism

The patient was placed in a supine position, and after successful induction of general anesthesia, routine disinfection and draping were performed. The right femoral artery was selected as the puncture site, and an 8F arterial sheath was inserted. With the support of a 6F guiding catheter, a contrast catheter was advanced to the internal carotid or vertebral artery. After heparinization, 3D angiography was performed to clearly delineate the aneurysm’s morphological features, including its shape, location, size, and the condition of the parent artery. Once the guiding catheter was positioned, the contrast catheter was withdrawn, and the microcatheter was shaped. Under the guidance of a microwire, the microcatheter was navigated into the aneurysm cavity. Based on the aneurysm’s size, an appropriate coil was selected and gradually packed into the aneurysm cavity through the microcatheter until dense packing was achieved. If necessary, stent assistance was employed to complete the endovascular embolization treatment. After surgery, all patients were admitted to the ICU for postoperative care and monitoring.

### The calculation of SAH volume

**Figure 2:**
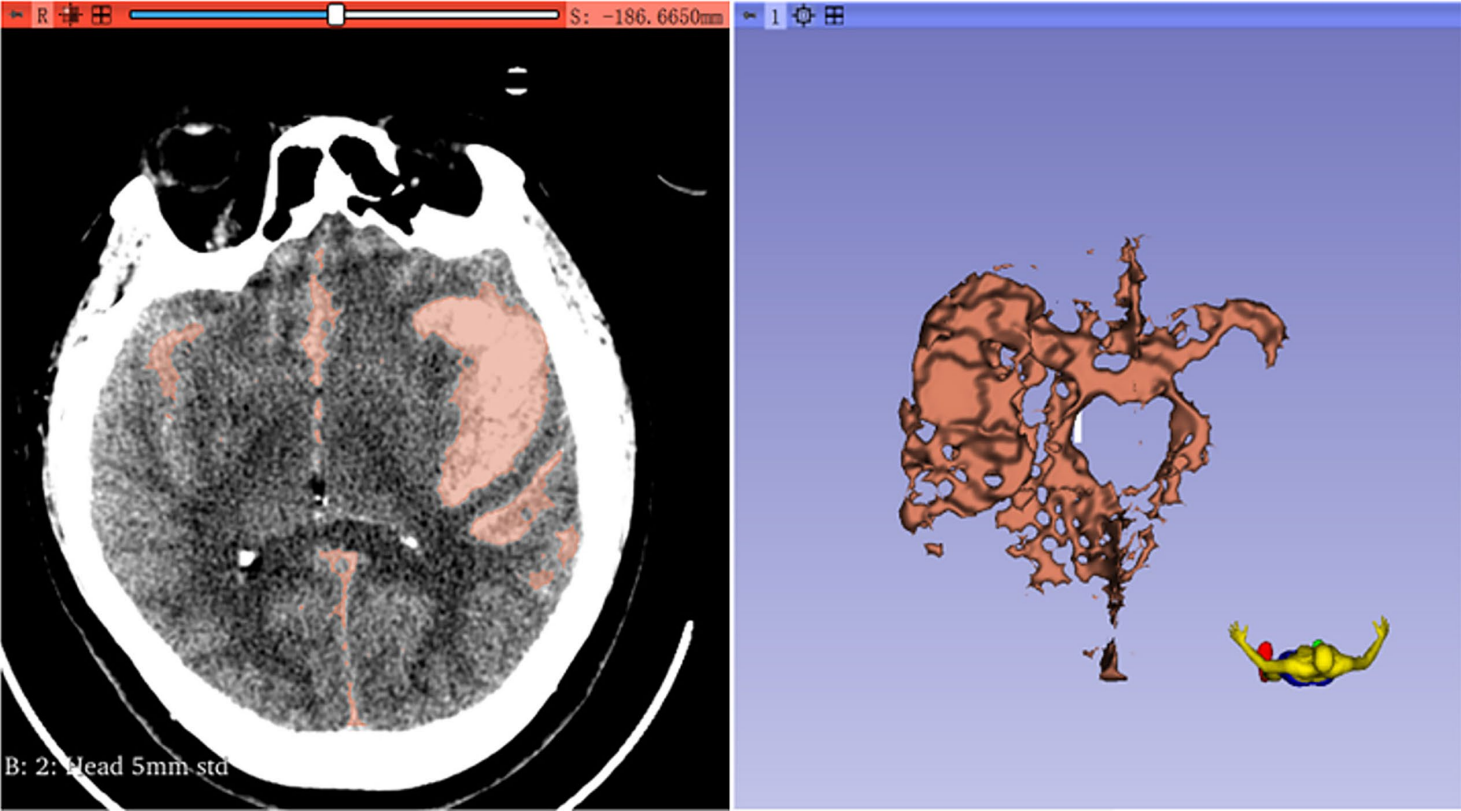
The process of intracerebral hemorrhage was calculated by 3D slice.

First, the volumes module is used to adjust the window width and window level, aiming to enhance the resolution between the hematoma and the surrounding tissues. Next, the Threshold method in the SegmentEditor module is employed to define the intensity range of the hematoma. The hematoma threshold is generally measured between 50 and 70 Hounsfield Units (HU), contingent upon the type of brain parenchyma (8, 9). Subsequently, the Paint and Erase functions are utilized to reconstruct the hematoma model. The Segmentations module is then used to convert the model into a 3D representation, and the hematoma volume is automatically calculated within the Models module (Figure 2). The rate of change in intracerebral hematoma volume [(HVpre-HVpost)/HVpre] is calculated by dividing the difference between the preoperative intracerebral hematoma volume (HVpre) and the postoperative volume at 6–8 days (HVpost) by the preoperative volume.

### Statistical analysis

Statistical analysis was conducted using SPSS 26.0 software. Frequency (percentage) [*n*(%)] was used to express count data, and the *χ*^2^ test was used for group comparisons. Measurement data with a normal distribution were expressed as mean ± standard deviation (*x* ± *s*), and group comparisons were performed using the two-sample t-test and one-way ANOVA, with further pairwise comparisons using the SNK-q method. Ordinal data were compared using the rank-sum test. The interquartile range was used to divide (HVpre-HVpost)/HVpre into four quartiles for stratified analysis to investigate the relationship between changes in cerebral hemorrhage and clinical outcomes. Factors identified in univariate analysis were included in a multivariate logistic regression model to determine independent risk factors affecting aSAH prognosis. The receiver operating characteristic (ROC) curve and precision recall (PR) curve was used to assess the predictive value of HVpre and HVpost for adverse outcomes. A *p*-value <0.05 was considered statistically significant.

## Results

This study included 62 patients (24 males [38.7%] and 38 females [61.3%]), with an average age of 57.79 ± 13.18 years. In terms of prognosis, 37 patients (59.68%) had a favorable outcome, while 25 patients (40.32%) had a poor outcome. Statistical analysis revealed significant differences between the good and poor prognosis groups in age, Glasgow Coma Scale (GCS), Hunt-Hess, mFS, HVpre, HVpost, and (HVpre−HVpost)/HVpre (*p* < 0.05) (Table 1).

**Table 1:** Demographics and clinical features of patients with aSAH.

Factor	All patients (*N* = 62)	Favorable outcome (*n* = 37)	Poor outcome (*n* = 25)	*P*-value
Gender				0.864
Male	24	14(37.8%)	10 (37.0%)	
Female	38	23 (62.2%)	15 (63.0%)	
Age (years)	57.79 ± 13.18	53.62 ± 10.43	63.96 ± 14.57	<0.05
Smoking	28	15 (40.54%)	13 (52.0%)	0.343
Alcohol	26	16 (43.24%)	10 (40.0%)	0.800
Hypertension	33	16 (43.24%)	17 (68.0%)	0.055
Diabetes	19	9 (24.32%)	10 (40.0%)	0.189
GCS				<0.05
13–15	39	28 (75.68%)	11 (44.0%)	
9–12	18	8 (21.62%)	10 (40.0%)	
3–8	5	1 (2.70%)	4 (16.0%)	
Hunt and Hess				<0.05
I	2	1 (2.70%)	1 (4.0%)	
II	43	31 (83.79%)	12 (48.0%)	
III	13	4 (10.81%)	9 (36.0%)	
IV	4	1 (2.70%)	3 (12.0%)	
mFisher				<0.05
0	1	1 (2.70%)	0 (0%)	
1	7	6 (16.22%)	1 (4.0%)	
2	30	22 (59.46%)	8 (32.0%)	
3	20	7 (18.92%)	13 (52.0%)	
4	4	1 (2.70%)	3 (12.0%)	
Aneurysm site				0.798
Anterior circulation	41	24 (64.87%)	17 (68.0%)	
Aneurysm size ≥ 10 mm	15	8 (21.62%)	7 (28.0%)	0.565
No. of aneurysms				0.702
1	51	31	20	
≥2	11	6	5	
Lumbar drainage	27	13	14	0.104
External ventricular drain	11	4	7	0.082
HVpre		22.68 ± 10.84	37.88 ± 10.03	<0.01
HVpost		15.24 ± 7.44	29.19 ± 8.69	<0.01
(HVpre-HVpost)/HVpre		0.355 ± 0.078	0.0234 ± 0.046	<0.05

Multivariate logistic regression analysis identified gender, age, HVpre, HVpost, and (HVpre−HVpost)/HVpre as independent risk factors influencing prognosis in aSAH patients (Table 2).

**Table 2:** Multivariate logistic regression analysis of prognostic factors after aSAH.

Factor	*B*	SE	Wals	Odds ratio	95% CI	*P*-value
Gender	−2.43	0.789	9.455	0.880	0.19–4.15	0.02
Age	0.181	0.051	13.165	1.203	1.09–1.33	0.01
Smoking	−0.284	0.773	0.135	0.753	0.17–3.43	0.713
Alcohol	−0.327	0.717	0.207	0.721	0.18–2.94	0.649
Hypertension	1.085	0.941	1.328	2.959	0.47–18.73	0.249
Diabetes	0.732	0.877	0.697	2.080	0.37–11.60	0.404
GCS	0.547	1.028	0.283	1.729	0.23–12.97	0.595
Hunt and Hess	−1.658	1.088	2.320	0.91	0.23–1.61	0.128
mFisher	3.259	1.034	9.932	2.604	0.34–19.67	0.102
Aneurysm site	0.922	0.689	1.790	2.515	0.65–9.71	0.181
Aneurysm size	1.078	0.892	1.459	2.939	0.51–16.90	0.227
No. of aneurysms	−0.733	0.893	0.674	0.480	0.08–2.77	0.412
Lumbar drainage	−0.624	0.895	0.486	0.536	0.09–3.10	0.486
HVpre	−0.294	0.515	0.325	0.746	0.27–2.04	0.037
HVpost	0.518	0.707	0.537	1.679	0.42–6.72	0.046
(HVpre-HVpost)/HVpre	−1.511	2.139	0.519	1.024	0.28–4.35	0.025

When comparing HVpre and HVpost with commonly used clinical scores, it was found that as GCS decreased and Hunt-Hess and mFS increased, HVpre and HVpost showed a statistically significant increase (*p* < 0.01) (Table 3).

**Table 3:** Relationship between preoperative and postoperative hematoma volume and clinical scoring scale in patients with aSAH.

Factor	n	HVpre	*P*-value	HVpost	*P*-value
GCS			<0.01		<0.01
13–15	39	21.48 ± 9.34		14.89 ± 7.57	
9–12	18	39.95 ± 7.13		29.60 ± 7.25	
3–8	5	45.84 ± 5.71		34.84 ± 5.71	
Hunt and Hess					<0.01
I	2	18.17 ± 13.31		12.73 ± 10.96	
II	43	25.99 ± 12.62		18.60 ± 10.45	
III	13	41.31 ± 8.61		30.66 ± 8.41	
IV	4	44.41 ± 3.98		33.93 ± 4.33	
mFisher					<0.01
0	1	8.76		5.12	
1	7	18.09 ± 6.70		10.78 ± 5.35	
2	30	20.30 ± 7.55		12.37 ± 6.34	
3	20	40.99 ± 6.26		30.45 ± 6.99	
4	4	43.44 ± 5.01		32.02 ± 5.37	

Stratified analysis of (HVpre−HVpost)/HVpre indicated that a higher hemorrhage reduction rate was associated with a lower likelihood of adverse clinical outcomes (Table 4).

**Table 4:** Outcome association with stratified analysis of the cerebral hemorrhage reduction.

Factor	(HVpre-HVpost)/HVpre < 22%	22 < (HVpre-HVpost)/HVpre < 29%	29 < (HVpre-HVpost)/HVpre < 39%	(HVpre-HVpost)/HVpre > 39%	*P*-value
Prognosis					0.008
Favorable outcome	5	8	11	13	
Poor outcome	11	8	4	2	

**Figure 3:**
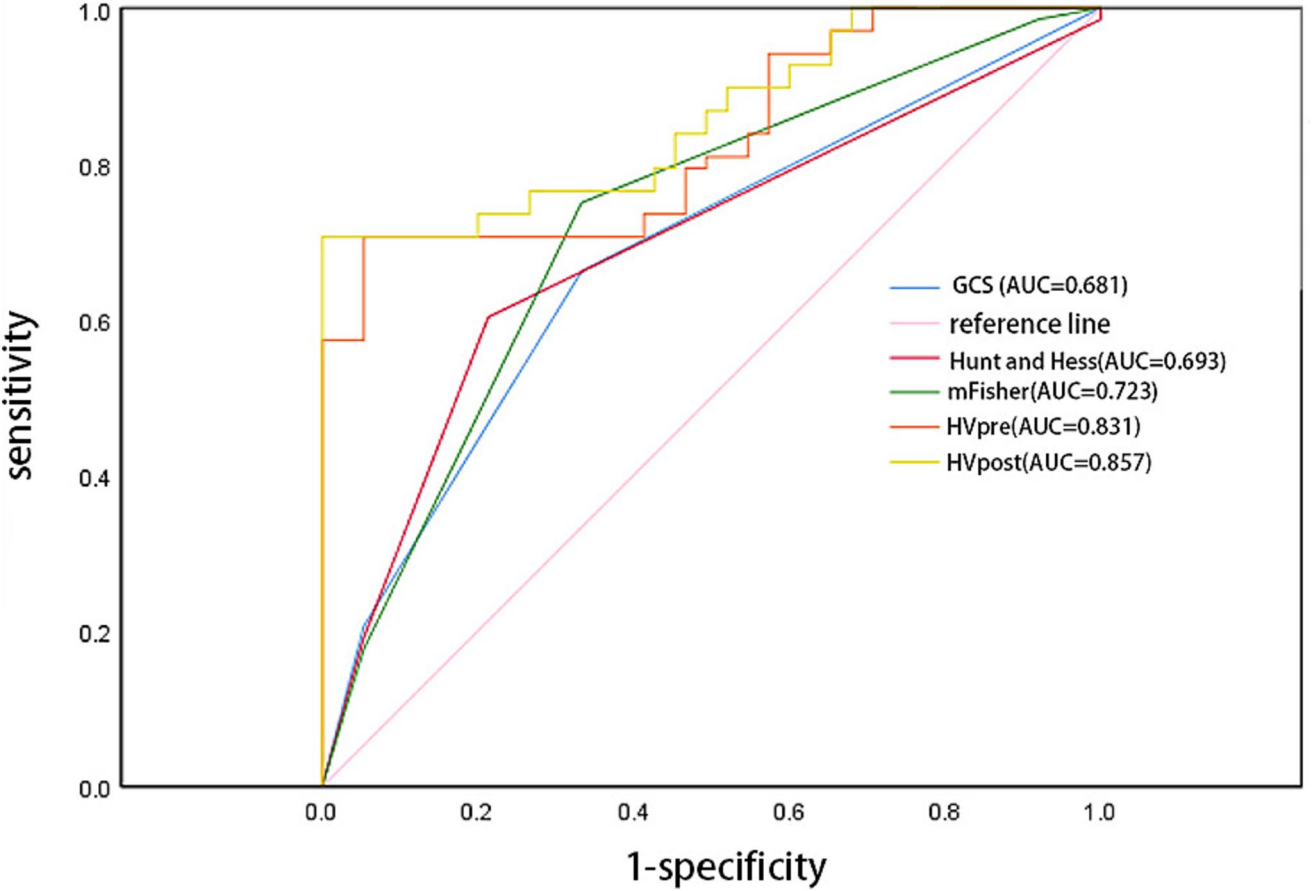
Comparison of several score models comparison using receiver operator characteristic curve.

**Figure 4:**
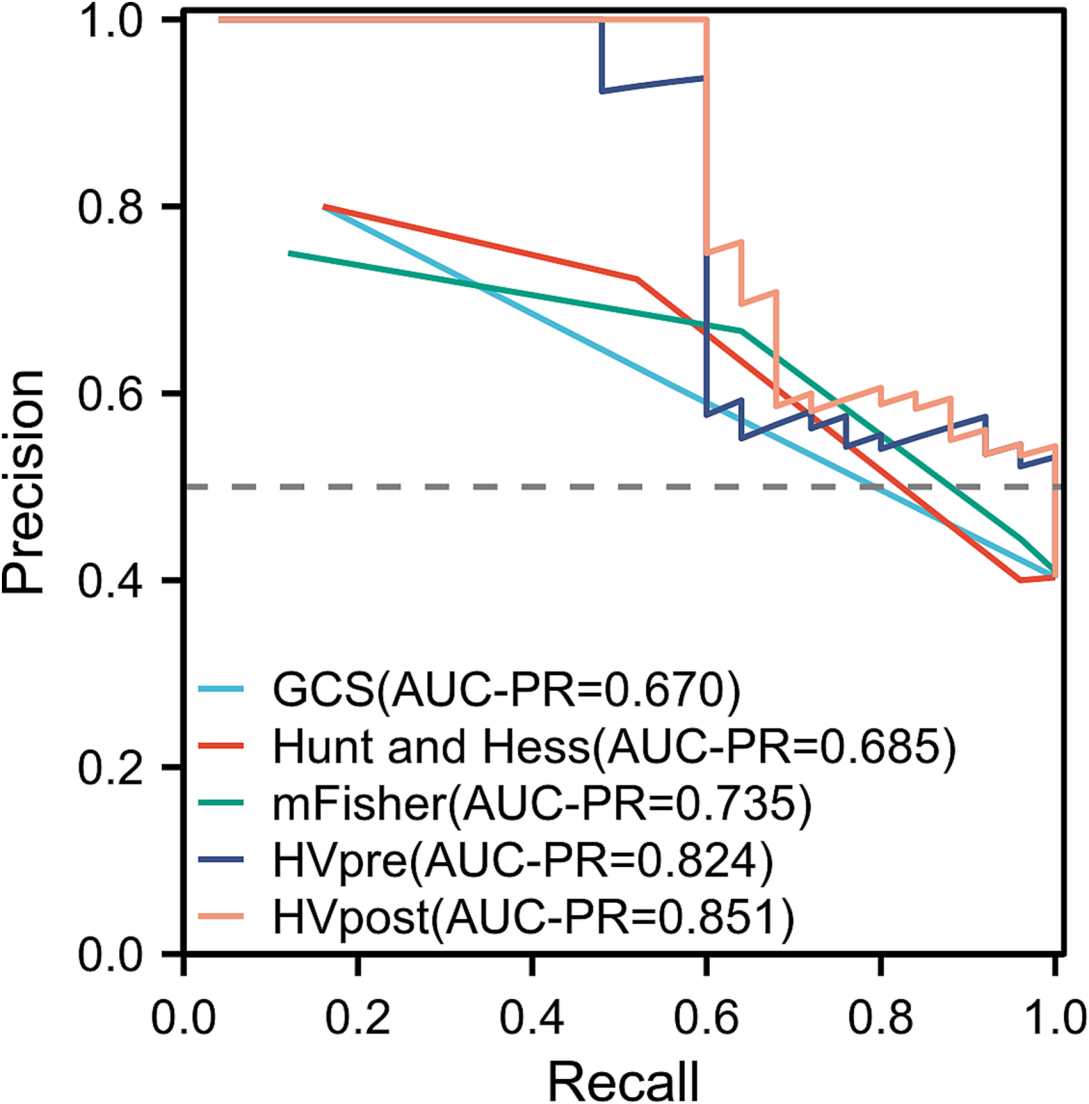
Comparison of several score models comparison using precision recall curve.

The ROC curve analysis showed that the AUC for GCS (0.681; 95% confidence interval [CI]: 0.593–0.769; *p* = 0.004), Hunt-Hess (0.693; 95% confidence interval [CI]: 0.605–0.782; *p* = 0.004), and mFS (0.723; 95% confidence interval [CI]: 0.639–0.807; *p* < 0.001) were similar. However, the AUCs for HVpre (0.831; 95% confidence interval [CI]: 0.763–0.899; *p* = 0.002) and HVpost (0.857; 95% confidence interval [CI]: 0.795–0.920; *p* < 0.001) were significantly higher than those for commonly used clinical scales. When HVpre volume exceeded 22.25 mL, the risk of poor prognosis increased significantly, with a sensitivity of 79.3% and a specificity of 67.1%. Similarly, when HVpost volume exceeded 15.67 mL, the risk of poor prognosis increased significantly, with a sensitivity of 80.7% and a specificity of 69.3% (Figure 3). At the same time, the PR curve results showed that the AUC of HVpre and HVpost were also higher than those of the commonly used clinical scales (Figure 4).

## Discussion

As populations age and lifestyles change, the global healthcare system is under increasing pressure, with stroke posing a significant threat to quality of life and life expectancy. aSAH, a subtype of stroke, has a high mortality and disability rate, with most survivors experiencing neurological dysfunction, which profoundly impacts both patients and their families (10). The volume of subarachnoid hemorrhage is closely linked to treatment choices and patient prognosis. Hemorrhage volume is now recognized as an independent predictor of delayed cerebral ischemia (DCI), seizures, and shunt-dependent hydrocephalus. It is also a critical indicator in determining the prognosis of aSAH patients, closely associated with clinical treatment strategies, functional recovery, and mortality. Therefore, accurate measurement of hematoma volume is of significant clinical importance (11, 12).

Previous studies have predominantly relied on clinical and radiological scores to assess the clinical symptoms and hematoma volume in aSAH, aiming to predict the likelihood of adverse outcomes and, consequently, determine prognosis (13). Clinical scores are mainly used to evaluate a patient’s mental status and neurological dysfunction, providing insight into the extent of brain injury following aSAH, as measured by scales such as GCS, Hunt-Hess, and mFS. Radiological scores, on the other hand, assess disease severity through imaging-based quantitative indicators, including hemorrhage volume, hemorrhage location, and the degree of brain edema observed on CT scans (14).

The Fisher scale or mFS is commonly used to assess aSAH through radiological scoring, predicting the occurrence of vasospasm and DCI by quantifying hemorrhage volume. However, the application of these scales remains controversial. A meta-analysis reviewing nearly 40 years of literature found that although both the Fisher scale and mFS can predict DCI by evaluating aSAH hematoma volume, the Fisher scale does not show a progressive relationship where higher grades consistently correspond to an increased likelihood of DCI, limiting its clinical utility (15). While the mFS partially addresses this limitation, a three-year single-center retrospective cohort study by Couret et al. (16) involving 230 aSAH patients revealed that the mFS has poor sensitivity and specificity in predicting vasospasm or DCI. Additionally, studies have highlighted that these scores are subject to individual variability, with the same CT images potentially yielding different results depending on the evaluator, resulting in only moderate inter-rater consistency for mFS scores (17). To address these limitations, Eagles et al. (18) introduced an innovative approach using a modified Graeb scale for semi-quantitative assessment of intraventricular hemorrhage following intracranial aneurysm rupture, replacing the qualitative description of intraventricular hemorrhage in the mFS. This new binary semi-quantitative evaluation scale has shown some improvement in predicting adverse outcomes. However, as a semi-quantitative system, it still relies on subjective evaluation of CT images by the reviewers, which does not resolve the issue of inter-rater reliability. Thus, we began to explore whether an objective and quantitative description of subarachnoid hemorrhage, intraventricular hemorrhage, and intracerebral hematoma before surgery and approximately 1 week after intracranial aneurysm rupture could address these issues and further improve the ability to predict prognosis.

Previous studies have suggested that the Tada formula is suitable for calculating hematomas that are relatively regular and ellipsoidal in shape. However, for subarachnoid hemorrhage, which often involves widespread bleeding and is frequently accompanied by ventricular system hemorrhage, the Tada formula is not appropriate (19). With the ongoing promotion of “precision medicine” in neurosurgery, accurately calculating the volume of irregular hematomas, which is crucial for patient treatment, has become an emerging research focus. 3D-Slicer, an open-source software that has recently gained attention for its role in surgical assistance, allows for the reconstruction of human tissues and organs from raw DICOM format data. It is compatible with multiple operating systems, runs smoothly on personal computers, and is relatively easy to use (20). Previous research has shown that 3D-Slicer can quantify the irregularity of spontaneous intracerebral hemorrhages, thereby predicting hematoma growth (21), and is particularly useful in the diagnosis and treatment of hypertensive intracerebral hemorrhage (22). However, there have been few studies assessing aSAH approximately 1 week after onset. Fragata et al. (23)noted that brain CT perfusion decreases 8–10 days after acute subarachnoid hemorrhage in DCI patients, which may have some value in preventing DCI. Therefore, this study utilized 3D Slicer to measure hematoma volumes in aSAH patients before surgery and 6–8 days post-surgery, and compared these measurements with commonly used clinical scales to evaluate their predictive value for patient prognosis. The goal is to provide an objective, precise, and quantitative method for assessing aSAH hematoma volume, thereby improving the accuracy of prognosis prediction.

This study found that age, GCS, and Hunt-Hess in aSAH patients were associated with prognosis. Additionally, mFS, HVpre, HVpost, and (HVpre−HVpost)/HVpre also demonstrated statistical significance in relation to prognosis. Building on these findings, further analysis using a multivariate logistic regression model revealed that HVpre, HVpost, and (HVpre−HVpost)/HVpre remained statistically significant predictors of prognosis and were identified as independent risk factors.

This study further examined the differences in HVpre and HVpost among patients with varying GCS, Hunt-Hess, and mFS, revealing that the overall differences in HVpre and HVpost across these groups were statistically significant (*p* < 0.05). While significant differences in HVpre and HVpost were observed between the higher (grades3 and 4) and lower (grades1 and 2) mFS levels, the differences in preoperative and approximately one-week postoperative hematoma volumes within the higher and lower levels themselves were minimal. This may help explain the discrepancies noted by Said et al. (14), where evaluators using mFS to assess the same imaging data had differing interpretations: when hematoma volumes are similar between grades, and since aSAH-related hemorrhage often spreads diffusely through the cisterns rather than being confined to a specific segment of the subarachnoid space, it becomes difficult for evaluators to clearly distinguish between adjacent grades based on visual assessment alone. For instance, it may be challenging to differentiate between mFS grade 3 and grade 4, or between grade 1 and grade 2.

In the stratified analysis of hematoma volume change rates, patients with greater changes in hematoma volume during the course of the disease had a lower likelihood of poor prognosis compared to those in the lower quartiles. Higher quartile values were associated with better clinical outcomes relative to the lowest quartile. ROC curve analysis indicated that both HVpre and HVpost have predictive value for poor prognosis, with HVpost showing a higher AUC (0.857 > 0.831), suggesting that HVpost has greater predictive value than HVpre. The PR curve shows the same result. Additionally, we found that when HVpre volume exceeds 22.25 mL, the risk of poor prognosis significantly increases, with a sensitivity of 79.3% and specificity of 67.1%. Similarly, when HVpost volume exceeds 15.67 mL, the risk of poor prognosis significantly increases, with a sensitivity of 80.7% and specificity of 69.3%. These findings suggest that HVpre, HVpost, and (HVpre−HVpost)/HVpre are reliable indicators of prognosis in aSAH patients.

This study has some limitations. First, it only compared hematoma volumes at preoperative and 6–8 days postoperative time points, without examining additional time points. Second, as a single-center retrospective study, it had a limited sample size and observation period, leading to a smaller number of patients in certain subgroups. Future research will involve larger-scale prospective studies to further elucidate the patterns of hematoma volume changes throughout the course of aSAH.

## Conclusion

HVpre, HVpost, and (HVpre−HVpost)/HVpre can serve as neuroimaging biomarkers for assessing patients after aSAH and can effectively predict clinical prognosis.

## Data Availability

The original contributions presented in the study are included in the article/supplementary material, further inquiries can be directed to the corresponding author.
